# Differentiating Hepatic Epithelioid Angiomyolipoma From Hepatocellular Carcinoma and Focal Nodular Hyperplasia via Radiomics Models

**DOI:** 10.3389/fonc.2020.564307

**Published:** 2020-10-06

**Authors:** Wenjie Liang, Jiayuan Shao, Weihai Liu, Shijian Ruan, Wuwei Tian, Xiuming Zhang, Dalong Wan, Qiang Huang, Yong Ding, Wenbo Xiao

**Affiliations:** ^1^Department of Radiology, College of Medicine, The First Affiliated Hospital, Zhejiang University, Hangzhou, China; ^2^Polytechnic Institute, Zhejiang University, Hangzhou, China; ^3^Department of Radiology, The People's Hospital of Beilun District, Ningbo, China; ^4^College of Information Science and Electronic Engineering, Zhejiang University, Hangzhou, China; ^5^Department of Pathology, College of Medicine, The First Affiliated Hospital, Zhejiang University, Hangzhou, China; ^6^Department of Hepatobiliary and Pancreatic Surgery, College of Medicine, The First Affiliated Hospital, Zhejiang University, Hangzhou, China

**Keywords:** hepatic epithelioid angiomyolipoma, hepatocellular carcinoma, focal nodular hyperplasia, radiomics, machine learning

## Abstract

**Background:** We conduct a study in developing and validating two radiomics-based models to preoperatively distinguish hepatic epithelioid angiomyolipoma (HEAML) from hepatic carcinoma (HCC) as well as focal nodular hyperplasia (FNH).

**Methods:** Totally, preoperative contrast-enhanced computed tomography (CT) data of 170 patients and preoperative contrast-enhanced magnetic resonance imaging (MRI) data of 137 patients were enrolled in this study. Quantitative texture features and wavelet features were extracted from the regions of interest (ROIs) of each patient imaging data. Then two radiomics signatures were constructed based on CT and MRI radiomics features, respectively, using the random forest (RF) algorithm. By integrating radiomics signatures with clinical characteristics, two radiomics-based fusion models were established through multivariate linear regression and 10-fold cross-validation. Finally, two diagnostic nomograms were built to facilitate the clinical application of the fusion models.

**Results:** The radiomics signatures based on the RF algorithm achieved the optimal predictive performance in both CT and MRI data. The area under the receiver operating characteristic curves (AUCs) reached 0.996, 0.879, 0.999, and 0.925 for the training as well as test cohort from CT and MRI data, respectively. Then, two fusion models simultaneously integrated clinical characteristics achieved average AUCs of 0.966 (CT data) and 0.971 (MRI data) with 10-fold cross-validation. Through decision curve analysis, the fusion models were proved to be excellent models to distinguish HEAML from HCC and FNH in comparison between the clinical models and radiomics signatures.

**Conclusions:** Two radiomics-based models derived from CT and MRI images, respectively, performed well in distinguishing HEAML from HCC and FNH and might be potential diagnostic tools to formulate individualized treatment strategies.

## Introduction

Preoperative evaluation of liver tumors sometimes remains a challenge for clinicians. On the one hand, there are still a large number of new hepatocellular carcinoma (HCC) cases each year due to the large population of hepatitis-B-related cirrhosis in China (1). On the other hand, with the increasing popularity of health screening, various types of hepatic masses have been asymptomatically detected. Clinicians need to evaluate plenty of hepatic lesions to implement individualized diagnosis, treatments and follow-up strategies for the patients. Hepatic epithelioid angiomyolipoma (HEAML) is an uncommon potential malignant tumor that belongs to the PEComas family, and it is pathologically characterized by perivascular epithelioid cell differentiation (2). As a special subtype of angiomyolipoma, HEAML without visible fat is easily confused with other blood-rich hepatic masses, including HCC and focal nodular hyperplasia (FNH) (3). Therefore, it is vital to precisely distinguish HEAML from non-HEAML hepatic lesions because diagnostic evaluation is an important prerequisite for implementing individualized treatment strategies. For HEAML, local surgical resection is ideal, despite there is a low proportion of tumor recurrence (3). According to the diagnosis and treatment guidelines, patients with HCC can individually undergo radiation therapy, surgical resection or transarterial chemoembolization after overall clinical evaluation. FNH usually only requires regular observation due to its completely benign biological behavior.

Previous imaging studies have observed that HEAML has specific radiological characteristics that may help with diagnostic evaluation on computed tomography (CT) and magnetic resonance imaging (MRI) (4–12). Definite existence of a small amount of fat in hepatic mass is greatly valuable in radiological diagnosis of HEAML, for which MRI scan is recommended (8, 10). Also, HEAML as a blood-rich tumor would be included in the differential diagnosis when draining vein of the hepatic mass was observed in the arterial phase (6, 8). In contrast to the wash-in and wash-out pattern of HCC, HEAML may have persistently high enhancement of the intertumoral focal area on contrast-enhanced CT or MRI (4–12). Although previous studies have explored the differential radiological characteristics of HEAML and HCC, no research has focused on the diagnostic evaluation to distinguish HEAML from FNH. Additionally, these radiological characteristics are usually morphological and non-quantitative, which rely on the observer's professional experience. Until now, the radiological diagnosis of HEAML has remained a clinical challenge.

In recent years, radiomics has become an active topic of medical artificial intelligence research. Previous studies have shown that high-throughput radiomics features extracted from medical imaging data can well predict tumor phenotypes (13). In the evaluation of liver tumors, especially HCC, radiomics can be used for tumor detection, evaluation of stage, treatment strategy selection, and prognosis prediction. Also, a small number of studies have shown that radiomics has potential predictive value for tumor classification (14). At first, Raman et al. (15) found the differentially expressed texture features in HCC, focal nodular hyperplasia and hepatic adenomas could be used differential diagnosis of these blood-rich lesions. Subsequently, deep learning method was used to classify liver masses using contrast-enhanced CT data (16). The CNN-based model showed excellently predictive efficiency in distinguishing malignant liver tumors from the non-malignant with an accuracy of 0.84 (16). A recent study also showed that deep learning model based on MRI data was a potential diagnostic tool for liver tumors (17).

Therefore, in this study, we tried to construct quantitative radiomics signature models for diagnosis of HEAML using CT and MRI images. Several classical machine learning algorithms have been tried to find the ideal model to classify HEAML and non-HEAML lesions. As far as we known, it was the first study based on a radiomics method to distinguish HEAML from other hepatic masses.

## Materials and Methods

### Patients

The review boards of First Affiliated Hospital, College of Medicine, Zhejiang University, approved the study protocol, and waived the requirement of informed consent from patients. Our datasets including contrast-enhanced CT and MRI data were retrospectively obtained during June 2009 to June 2017 for this study. In detail, 170 patients with contrast-enhanced CT images (78 HCC, 59 FNH, 33 HEAML) and 137 patients with contrast-enhanced MRI images (77 HCC, 30 FNH, 30 HEAML) were enrolled. For both CT and MRI datasets, the patients diagnosed with HEAML were included in an HEAML group, and the patients with HCC or FNH constituted a non-HEAML group.

**Figure 1:**
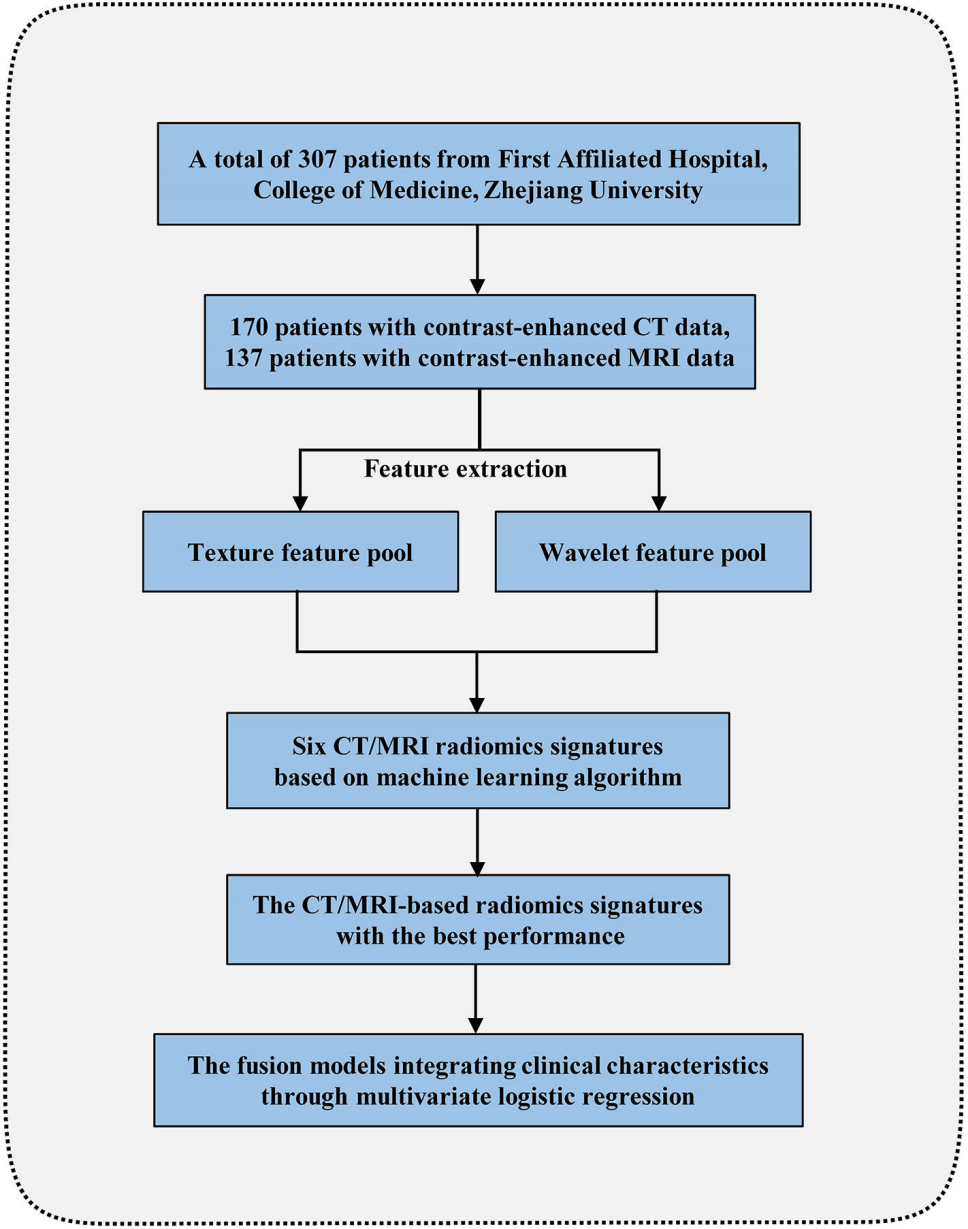
The flowchart for this radiomics study.

The inclusion criteria for the patients were as follows: (1) HEAML, HCC, and FNH diagnosed pathologically by surgical resection or biopsy; (2) contrast-enhanced CT or MRI scans performed within 1 month before operation; (3) complete imaging data for further analysis. Patients would be excluded due to the following criteria: (1) diagnosis of recurrent tumor or multiple organ malignant tumor; (2) antitumor treatment received before contrast-enhanced CT or MRI scan; (3) poor imaging quality of liver mass. The flow chart for our radiomics study is shown in Figure 1.

### Imaging Data Parameters

All patients underwent contrast-enhanced CT or MRI scans before surgery or biopsy. CT scans included multislice spiral CT (Aquilion 16, Toshiba Medical Systems, Otawara, Japan) and 256-slice CT (Brilliance iCT, Philips Medical Systems, Cleveland, USA). The scanning parameters were as follows: tube voltage 125 kVp; tube current 320 mAs; pitch 0.95 mm; layer thickness 2–5 mm; reconstruction interval 2 mm. The contrast agent used for enhanced CT was iohexol (Jiangsu Hengrui Pharmaceutical Co., Ltd., Lianyungang, China). The high-pressure syringe speed was 3.0 ml/s, the injection volume was 1.5 ml/kg, and the forelimb was injected intravenously. Dynamic enhanced scanning was performed at 25–30, 60–65, and 120–140 s after the contrast agent was injected during the arterial phase, portal vein phase as well as delayed phase.

MRI scans were performed using a high-field-strength MRI instrument (3.0 T Signa HDx, GE Medical Systems, WI, USA). The contrast-enhanced MRI scan sequence was the simultaneous liver acceleration volume acquisition sequence using breath gating. The image acquisition parameters were as follows: repeat time (TR) 3.3 ms; echo time (TE) 1.5 ms; flip angle 10°; matrix 320 ^*^ 256; layer thickness 5 mm. The contrast agent was gadolinium-diethylenetriamine penta-acetic acid (Gd-DTPA, Magnevist, Bayer HealthCare, Berlin, Germany). The injection rate was 2.0–3.0 ml/s, and the injection dose was 0.1 mmol/kg. Dynamic enhancement scanning was performed 15–20, 40–55, and 140–180 s after contrast agent injection.

### Region of Interest (ROI) Segmentation and Data Division

Contrast-enhanced CT/MRI arterial phase data were used for the radiomics analysis. ROIs were manually segmented using ITK-SNAP on a cross-sectional layer with a maximum diameter of mass with CT/MRI imaging data (simultaneously avoiding a large necrotic layer, Supplementary Figure 1). The segmentation was completed by an experienced radiologist and proofread by a senior radiologist.

### Radiomics Feature Extraction

Since the different voxel sizes always influence imaging features, we had to resample the images first to extract reproducible radiomics features (18, 19). Therefore, spline interpolation was used and the voxel intensities of each ROI image were discretized to a value of 64 bins (20).

After image resampling to the identical spatial voxel size and voxel intensities, we extracted quantitative texture features from original CT and MRI data. Additionally, the original image was decomposed by the Haar wavelet transform to obtain high-order wavelet features. Through changing the ratio of high-frequency to low-frequency signals and reconstructing images in different forms, every image was decomposed and reconstructed into 8 additional images. The size of the decomposed images was equal to that of the original image. From each reconstructed image, wavelet features were extracted. The radiomics features extracted in our study are stable and can be reproduced through the methods we introduced (20–22). Also, a filtering feature screening (mutual information) method was implemented to reduce the feature dimension.

### Construction and Evaluation of the Radiomics Signatures

For both CT and MRI datasets, significant imbalance on patient population existed between the HEAML group and the non-HEAML group. Therefore, synthetic minority over-sampling technique (SMOTE) was applied to balance population as well as avoid overfitting. The original datasets (HEAML group and non-HEAML group) were proportionally divided into a training cohort and a test cohort at the ratio of 2:1. The populations of HEAML patients and non-HEAML patients were set to be consistent in the training cohort and the test cohort. After the cohorts had been divided, SMOTE was alone implemented on the training cohort. In this way, we not only solved the problem of unbalanced samples, but also ensure the independence of test cohort for model evaluation.

To construct high-performance radiomics signatures, random forest (RF), artificial neural network (ANN) as well as ridge regression (RR), were separately applied to the training cohort and the test cohort. As a result, three radiomics signatures based on three different algorithms were constructed. The optimal parameters of each algorithm were obtained with a grid-search method. The receiver operating characteristic (ROC) curves were plotted, then the AUCs were calculated to estimate the efficiency of every radiomics signature. Finally, the selected radiomics signature model was used to construct a fusion model in the following steps. The operations above were conducted two times, and two high-performance radiomics signatures were eventually acquired based on CT and MRI datasets, respectively.

### Construction and Evaluation of the Fusion Models

It is assumed that information on clinical characteristics is of additional value to the differential diagnosis (23–25). Therefore, radiomics signatures and clinical characteristics were applied to construct fusion models based on the multivariate logistic regression (MLR) algorithm. In our study, clinical characteristics included sex, age, maximum diameter, tumor location, alcoholism, and smoking. Two steps should be followed to construct the fusion models. Firstly, according to the Akaike information criterion (AIC), the combined clinical characteristics as well as radiomics signature of the lowest AIC value were selected, acting as the components of the fusion model. Secondly, the 10-fold cross-validation was applied to establish fusion models, verifying the confidence of the results. In the process, SMOTE was not used in the building of the fusion models. The average AUCs of the fusion models based on CT and MRI datasets, respectively, were selected to show the diagnostic efficiency of the models. Meanwhile, we applied decision curve analysis (DCA) to confirm the improvement in the models after the clinical factors had been taken into consideration. Finally, to realize the application of the fusion models in clinical practice, diagnostic nomograms were built, which would help preoperatively distinguish HEAML from FNH and HCC.

### Statistical Analysis

The feature extraction program was conducted in MATLAB (2016a) (MathWorks, Natick, MA, USA). RF, RR, and ANN algorithms were conducted with python 3.7.0 (https://www.python.org/). The AUC was calculated and depicted by the “pROC” package. Diagnostic nomograms were built with the “rms” package in R software 3.6.2 (https://www.r-project.org/). The statistical results of continuous variables (including age and maximum diameter) were obtained based on a two-sided Mann–Whitney *U-*test. The statistical results of categorical variables (including sex, tumor location, alcoholism, and smoking) were acquired through a two-sided chi-squared test. The Mann-Whitney *U-*test and chi-square test were implemented by SPSS 20 (IBM Corp, Chicago, USA).

## Results

### Clinical Factors of Patients

According to the clinical records of the patients, five clinical characteristics, including sex, age, the maximum diameter, tumor location, alcoholism, and smoking, were selected as potential biomarkers for differential diagnosis. The statistical results of clinical characteristics between HEAML group and non-HEAML group are shown in Table 1.

**Table 1:** The statistical results of clinical characteristics between HEAML group and non-HEAML group.

	**CT**			**MRI**		
**Clinical Characteristics**	**HEAML (*n =* 28)**	**Non-HEAML (*n =* 128)**	***p***	**HEAML (*n =* 26)**	**Non-HEAML (*n =* 98)**	***p***
Sex			<0.001			<0.001
Male	6 (21%)	93 (73%)		7 (27%)	78 (80%)	
Female	22(79%)	35 (27%)		19 (73%)	20 (20%)	
Age	47.7 ± 10.4	45.6 ± 16.3	0.701	49.0 ± 9.8	50.4 ± 12.7	0.583
The Maximum Diameter	4.4 ± 1.8	5.3 ± 2.8	0.265	4.3 ± 2.2	5.1 ± 2.8	0.298
Tumor Location			<0.001			1.000
Left	25 (89%)	55 (43%)		10 (38%)	38 (39%)	
Right	3 (11%)	73 (57%)		16 (62%)	60 (61%)	
Alcoholism or Smoking			<0.001			0.014
No	25 (89%)	51 (40%)		21 (81%)	53 (54%)	
Yes	3 (11%)	77 (60%)		5 (19%)	45 (46%)	

*HEAML represents hepatic epithelioid angiomyolipoma. The values of age and the maximum diameter are shown as mean ± standard deviation. The clinical records of fourteen patients in CT dataset and thirteen patients in MRI dataset are partly incomplete*.

### Radiomics Feature Extraction

Totally, we extracted 423 quantitative radiomics features from the ROIs of CT or MRI data from each patient with HEAML, FNH, and HCC. There were three types of radiomics features in this study: 7 first-order histogram statistical features, 40 texture features, as well as 376 features using wavelet transform. The texture features included 5 features extracted from the neighborhood gray-tone difference matrix (NGTDM), 13 from the gray-level size zone matrix (GLSZM), 13 from the gray-level run-length matrix (GLRLM), and 9 from the gray-level cooccurrence matrix (GLCM). More details about the radiomics features extracted are available in Supplementary Table 1. After the pre-screening based on mutual information method, 80 CT radiomics features and 95 MRI radiomics features were selected for the construction of radiomics signatures.

### The Construction and Evaluation of the Radiomics Signatures

**Figure 2:**
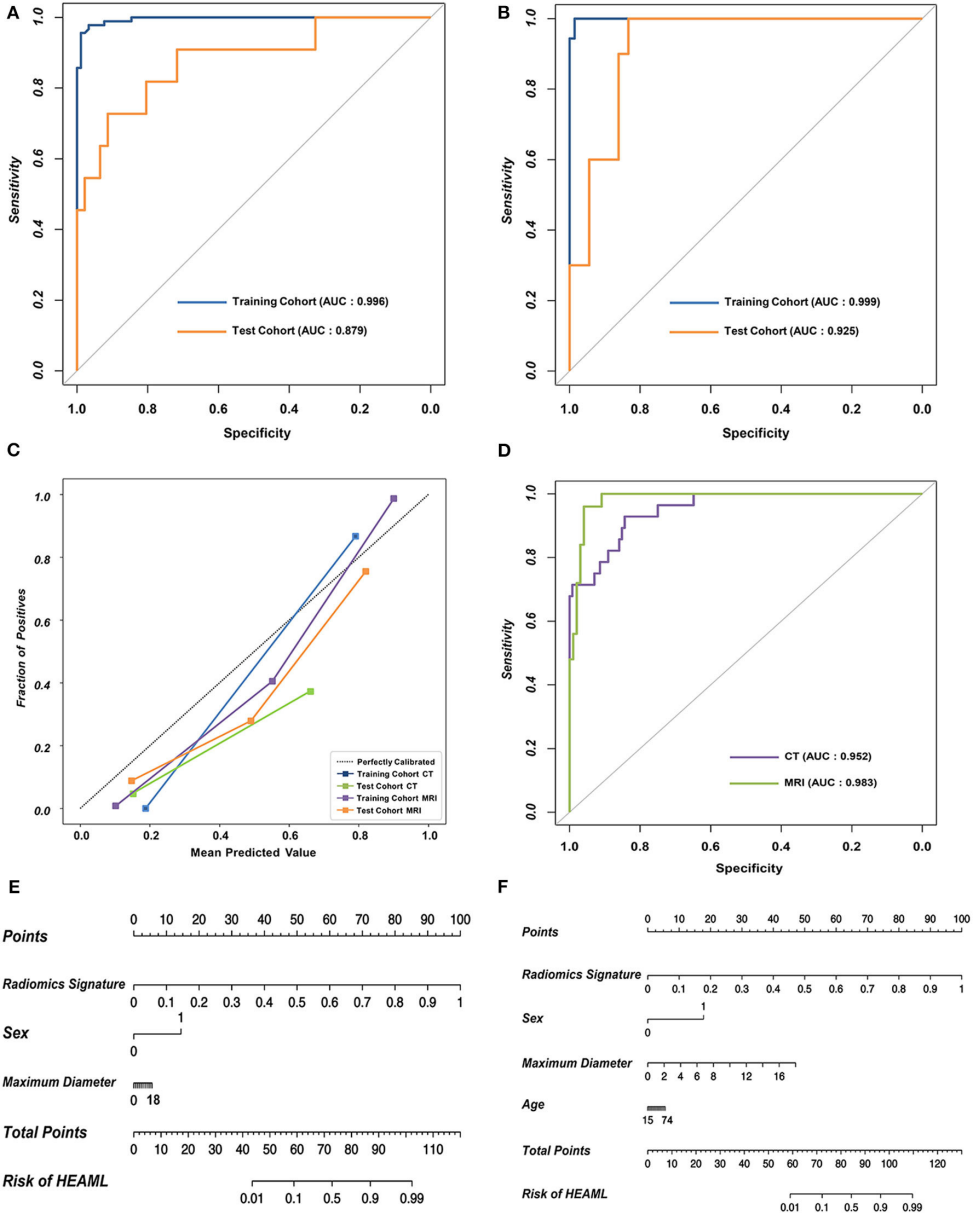
The performance of the RF-based radiomics signatures and fusion models integrating the radiomics signatures and clinical characteristics. **(A,B)** The ROCs of the two RF-based radiomics signatures with CT and MRI datasets, respectively. **(C)** The calibration curves revealing the performance of the proposed radiomics signatures. **(D)** The overall ROCs of the fusion models. **(E,F)** The diagnostic nomograms based on the fusion models.

As is shown in Table 2, the results of AUCs were listed based on three different machine learning algorithms. Results showed that the radiomics signatures based on the RF algorithm performed the best with both CT and MRI datasets. The AUCs reached 0.996, 0.879 for the training group as well as the test group from CT dataset, respectively, and were 0.999, 0.925 for the training group, test group from MRI dataset, respectively. The ROCs of the RF-based radiomics signatures are plotted in Figures 2A,B. Furthermore, the calibration curves showed that the predicted outcomes of RF-based radiomics signatures coordinated with the real diagnostic results (Figure 2C). It showed that the radiomics signatures constructed by RF were the optimal models. In addition, the radiomics features weights obtained during the construction of RF-based radiomics signatures are listed in Supplementary Table 2.

**Table 2:** The performance of radiomics signatures constructed by three machine learning algorithms and two datasets.

**Type of dataset**	**Algorithm**	**Training cohort**		**Test cohort**	
		**AUC**	**95% CI**	**AUC**	**95% CI**
CT	RR	0.907	0.867–0.947	0.731	0.572–0.891
	RF	0.996	0.991–1.000	0.879	0.752–1.000
	ANN	0.861	0.802–0.919	0.763	0.629–0.896
MRI	RR	0.997	0.994–1.000	0.736	0.523–0.949
	RF	0.999	0.997–1.000	0.925	0.851–0.999
	ANN	0.987	0.968–1.000	0.769	0.592–0.946

*RF, RR, and ANN represent random forest, ridge regression, artificial neural network, respectively. CT and MRI represent computed tomography and magnetic resonance imaging, respectively. AUC refers to the area under the curve. CI refers to confidence interval*.

### The Construction and Evaluation of the Fusion Models

The optimal combination of clinical characteristics and radiomics signature was determined according to the AIC values (Supplementary Table 3). The ROCs of two fusion models are plotted in Figure 2D. The fusion models achieved an average AUC of 0.966 with CT dataset and 0.971 with MRI dataset. The ROCs of clinical models are plotted (Supplementary Figure 2). According to the net benefit, the fusion models were superior over the radiomics signatures and clinical models at the overall level (Supplementary Figure 3). Finally, two diagnostic nomograms were built (Figures 2E,F) based on the fusion models.

## Discussion

CT/MRI radiomics signatures and the fusion models were developed separately and validated the prediction efficiency for HEAML diagnostic evaluation. The RF-based radiomics signature performed well with AUCs of 0.996, 0.879 for the training cohort, test cohort from CT dataset, respectively, and of 0.999, 0.925 for the training cohort, test cohort from MRI dataset. Furthermore, several clinical characteristics were included, and two high-performance fusion models were put forward. The fusion models outperformed the clinical models and radiomics signatures in the diagnostic prediction. The fusion models achieved an average AUC of 0.966 with CT dataset and 0.971 with MRI dataset. Our results showed that the radiomics features can potentially be used for the preoperative diagnosis of HEAML *vs*. HCC and FNH.

High-order radiomics features often play an important role as predictors in radiomics model studies (26–28). In a previous study, high-order radiomics features with deep learning methods were applied to the differential diagnosis of fatty liver diseases and liver tumors (14). Moreover, another study proposed a high-order feature-based radiomics model to differentiate liver masses from HCCs (16). The effective classification of HEAML and non-HEAML liver tumors demonstrated the quantitative radiomics features played an irreplaceable role in our study. Interestingly, part of these selected high-order features were related to coarseness, correlation, busyness, sum average and variance of the medical images. Because it was just a preliminary radiomics study, the biological information behind the selected features still needs to be further explored.

Age and sex were important clinical factors in our fusion models for diagnostic evaluation of HEAML *vs*. FNH and HCC. In a previous study, the average onset age of HEAML was ~51 years (7), while the average age was 56 years in another study (12). We believed this difference was due to the divergence of cases and the small sample size. Our study enrolled 28 cases of HEAML with an average age of 47.7 ± 10.4 years. Unlike HEAML, the onset age of the HCC group is usually older. However, the onset age of the FNH group was relatively younger. In addition, our results showed that HEAML and FNH usually occurred in females, and HCC tended to occur in males. Our results also showed that the clinical factors could improve the predictive performance of radiomics signature models. Therefore, age and sex were integrated in our fusion models to evaluate the possibility of HEAML.

Several studies have proven that CT-based and MRI-based radiomics features both have the ability to discriminate different tumor phenotypes (29–31). A study found that both CT-based and MRI-based radiomics models can detect lymph node metastases in cervical cancer (30). In addition, CT and MRI data can be applied to the preoperative evaluation of pancreatic cancer with excellent diagnostic efficiency (29). Our study found that the radiomics signatures and fusion models based on two different types of images were both highly efficient on the post-operative evaluation of HEAML. Moreover, the efficiency of radiomics signature and fusion model based on MRI images was slightly higher than the models based on CT images. We believe that the prediction models based multimodal imaging data will facilitate clinical use of individual diagnosis and treatment.

The advantages of this study are listed below. Previous research explored the morphological features of HEAML (4–12). However, the use of quantitative features to differentiate HEAML from other liver masses has not been reported using radiomics method. In this study, we used two radiomics-based models to distinguish HEAML from HCC and FNH with contrast-enhanced CT and MRI data. Higher-order features reflecting intratumor heterogeneity were used to build the radiomics signature models. Additionally, the prediction models of two types of imaging data were available for clinicians to use. Evidently, our results showed that the models constructed based on radiomics features were diagnostic tools for the classification of blood-rich hepatic lesions.

Our retrospective study also has some limitations. First, although we increased the number of patients over a long-time span, the number of patients with HEAML was still relatively small because HEAML is uncommon. Second, conventional imaging features were not included because this research focused on the efficiency of quantifying imaging features in the diagnostic evaluation of HEAML. In our follow-up work, conventional imaging features will be incorporated into the models to improve the efficiency of diagnosis. Third, 2D ROI data were used for model construction, which might be a disadvantage because 3D ROI data include more information about tumor heterogeneity. Later, different types of data (2D/3D) and different separation methods (manual/semiautomatic/fully automatic segmentation) will be considered in the next stage of our radiomics research.

In conclusion, this study proposed two CT/MRI-based radiomics models for the differential diagnosis of HEAML. The developed nomograms can be used for non-invasive preoperative evaluation of liver tumors, which will be helpful for the individual diagnosis and treatment of HEAML.

## Data Availability Statement

The raw data supporting the conclusions of this article will be made available by the authors, without undue reservation.

## Ethics Statement

The review boards of First Affiliated Hospital, College of Medicine, Zhejiang University, approved the study protocol and waived the requirement of informed consent from patients.

## Author Contributions

WLia, WLiu, XZ, and YD: conception and design. WX, JS, SR, and WT: data analysis and interpretation. WLia, WLiu, DW, and QH: manuscript writing. All authors: review and editing.

## Conflict of Interest

The authors declare that the research was conducted in the absence of any commercial or financial relationships that could be construed as a potential conflict of interest.
