# Supplementary material for: Differentiating Hepatic Epithelioid Angiomyolipoma From Hepatocellular Carcinoma and Focal Nodular Hyperplasia via Radiomics Models
Source: Front Oncol. 2020 Oct 6;10:564307. doi: 10.3389/fonc.2020.564307 (PMC7573543; doi:10.3389/fonc.2020.564307)
Supplement: Supplementary file 1 [file Data_Sheet_1.docx]

**Supplementary Materials**

**[Supplementary Figures](#_Toc46593412)** [2](#_Toc46593412)

**[Supplementary Tables](#_Toc46593413)** [5](#_Toc46593413)

**[Supplementary Formula](#_Toc46593414)** [9](#_Toc46593414)

**Supplementary Figures**

**
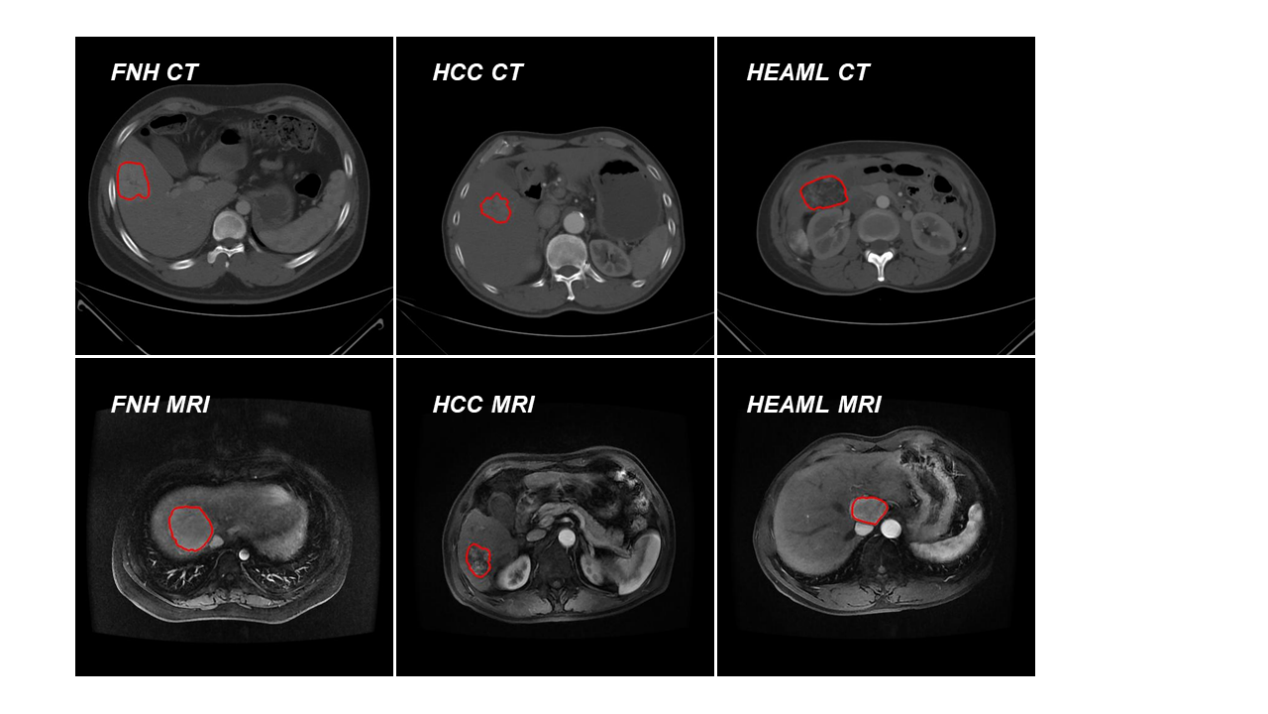
**

**Figure S1.** ROI segmentation of hepatic lesions.

**
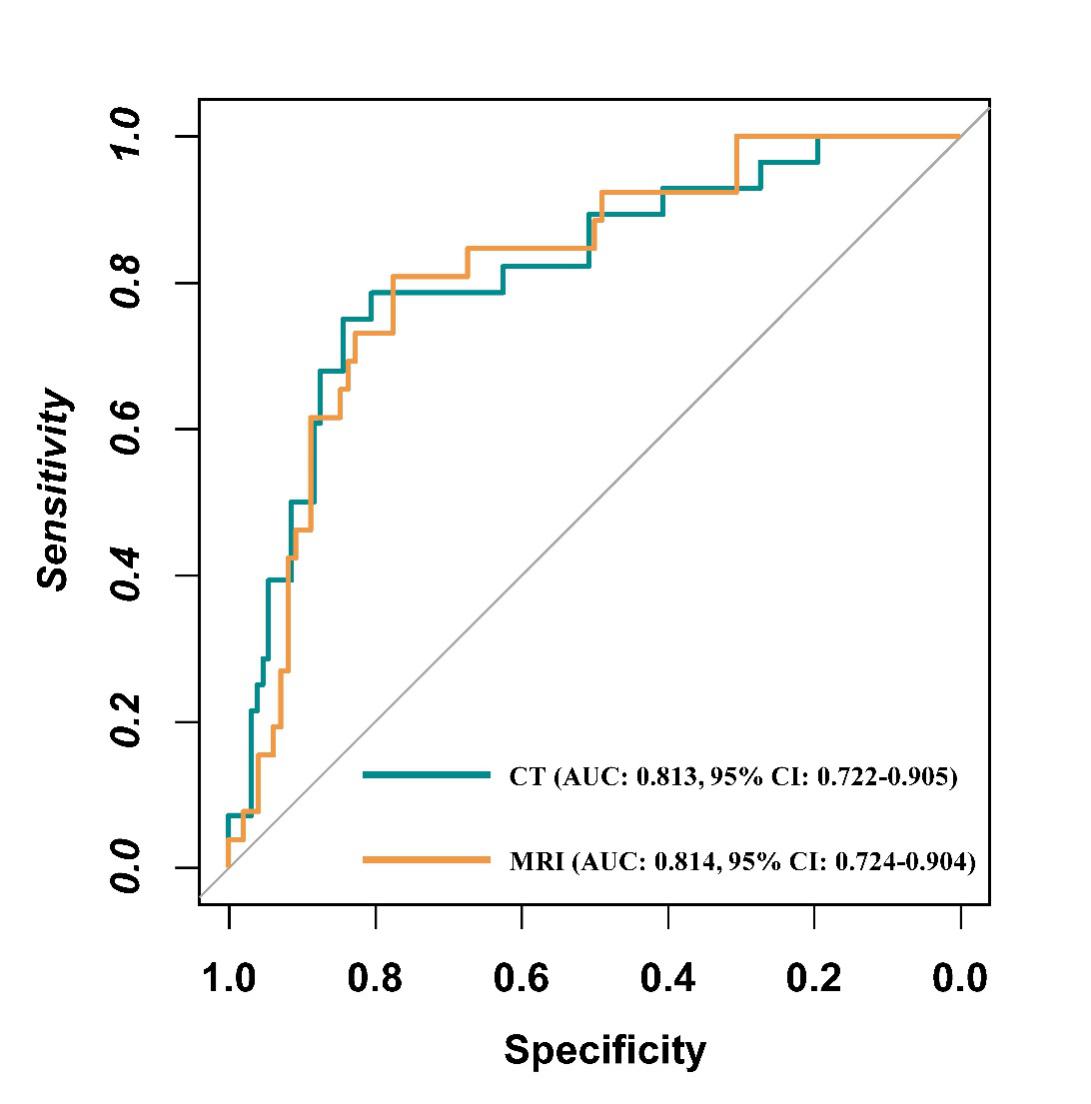
**

**Figure S2.** The ROCs of clinical models.

**
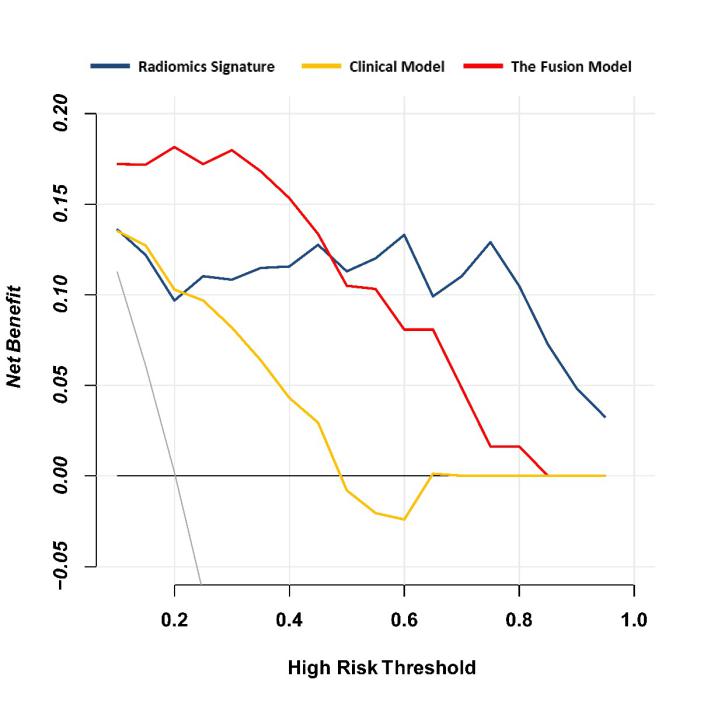
`**

**Figure S3.** The results of DCA showed the comparison among the clinical models, radiomics signatures and the fusion models respectively with MRI data.

**Supplementary Tables**

**Table S1:** **The radiomics features extracted in our study.**

| **Feature Category** | **Features in Detail** |
| --- | --- |
| Histogram features | Mean, Variance, Deviation, Skewness, Kurtosis, Energy, Entropy, |
| Texture features | Energy, Contrast, Entropy, Homogeneity, Correlation, SumAverage, Variance, Dissimilarity, AutoCorrelation derived from GLCM.  Short Run Emphasis (SRE), Long Run Emphasis (LRE), Gray-Level Nonuniformity (GLN), Run-Length Nonuniformity (RLN), Run Percentage (RP), Low Gray-Level Run Emphasis (LGRE), High Gray-Level Run Emphasis (HGRE), Short Run Low Gray-Level Emphasis (SRLGE), Short Run High Gray-Level Emphasis (SRHGE), Long Run Low Gray-Level Emphasis (LRLGE), Long Run High Gray-Level Emphasis (LRHGE), Gray-Level Variance (GLV), Run-Length Variance (RLV) derived from GLRLM.  Small Zone Emphasis (SZE), Large Zone Emphasis (LZE), Gray-Level Nonuniformity (GLN), Zone-Size Nonuniformity (ZSN), Zone Percentage (ZP), Low Gray-Level Zone Emphasis (LGZE), High Gray-Level Zone Emphasis (HGZE), Small Zone Low Gray-Level Emphasis (SZLGE), Small Zone High Gray-Level Emphasis (SZHGE), Large Zone Low Gray-Level Emphasis (LZLGE), Large Zone High Gray-Level Emphasis (LZHGE), Gray-Level Variance (GLV), Zone-Size Variance (ZSV) derived from GLSZM.  Strength, Busyness, Complexity, Contrast, Coarseness derived from NGTDM. |
| Wavelet features | Wavelet texture features derived from images transformed in low-frequency sub-bands, horizontal high-frequency sub-bands, vertical high-frequency sub-bands, and diagonal high-frequency sub-bands at successive multiscale. |

**Note:** GLCM, Gray-Level Co-occurrence Matrix; GLRLM, Gray-Level Run-Length Matrix; GLSZM, Gray-Level Size Zone Matrix; NGTDM, Neighborhood Gray Tone Difference Matrix (the same below in Table S2).

**Table S2. The weights of radiomics features constructing the RF-based radiomics signatures.**

| **CT** | | | | **MRI** | | | |
| --- | --- | --- | --- | --- | --- | --- | --- |
| **Feature Name** | **Weight** | **Feature Name** | **Weight** | **Feature Name** | **Weight** | **Feature Name** | **Weight** |
| GLSZM_LGZE  GLCM_Correlation  GLRLM_GLV  GLSZM_LZLGE  GLRLM_LRHGE  GLRLM_HGRE  GLCM_AutoCorrelation  NGTDM_Coarseness  GLSZM_LZHGE  grayFeature_entropy  grayFeature_energy  grayFeature_deviation  grayFeature_variance  GLRLM_SRE  GLRLM_LGRE  NGTDM_Strength  GLRLM_SRLGE  GLSZM_LGZE  GLCM_Correlation  NGTDM_Strength  GLSZM_GLN | 0.104  0.094  0.090  0.053  0.043  0.043  0.041  0.038  0.034  0.031  0.026  0.025  0.024  0.023  0.022  0.022  0.020  0.020  0.019  0.018  0.018 | GLRLM_RP  grayFeature_entropy  GLRLM_GLV  grayFeature_entropy  GLRLM_LRLGE  grayFeature_entropy  grayFeature_mean  grayFeature_variance  GLRLM_GLV  GLCM_SumAverage  grayFeature_deviation  grayFeature_energy  GLSZM_LZE  GLSZM_LGZE  GLSZM_SZLGE  NGTDM_Contrast  grayFeature_variance  GLRLM_RLV  GLRLM_RP  grayFeature_deviation | 0.018  0.018  0.016  0.015  0.013  0.013  0.012  0.011  0.010  0.010  0.010  0.009  0.009  0.009  0.007  0.005  0.003  0.002  0.002  0.001 | NGTDM_Strength  GLSZM_LGZE  GLSZM_LZLGE  NGTDM_Strength  GLCM_Energy  GLSZM_LZLGE  GLCM_SumAverage  GLSZM_GLN  GLCM_Correlation  GLRLM_LRLGE  NGTDM_Busyness  GLCM_SumAverage  GLCM_Correlation  GLCM_Variance  GLSZM_LZLGE  GLSZM_SZE  grayFeature_kurtosis  GLSZM_LZE  NGTDM_Contrast  GLRLM_LRHGE  NGTDM_Strength  GLCM_Correlation  GLCM_Homogeneity  grayFeature_skewness  GLSZM_LGZE  GLCM_Energy  grayFeature_kurtosis  GLCM_Entropy | 0.085  0.069  0.056  0.037  0.036  0.036  0.034  0.034  0.032  0.032  0.031  0.030  0.029  0.027  0.026  0.025  0.023  0.022  0.019  0.018  0.016  0.016  0.015  0.015  0.014  0.014  0.013  0.013 | GLRLM_HGRE  GLRLM_RP  NGTDM_Contrast  grayFeature_kurtosis  GLSZM_LGZE  GLRLM_LRE  GLSZM_SZHGE  GLSZM_LZLGE  GLCM_Correlation  GLRLM_SRHGE  GLSZM_ZSN  grayFeature_kurtosis  GLCM_SumAverage  GLSZM_LGZE  NGTDM_Busyness  GLCM_Variance  GLSZM_ZP  GLSZM_SZHGE  GLSZM_SZLGE  GLRLM_RLV  GLSZM_ZSN  grayFeature_kurtosis  GLSZM_SZE  GLSZM_ZP  GLRLM_GLN  GLCM_Dissimilarity  GLSZM_ZP  GLSZM_SZHGE | 0.013  0.012  0.012  0.012  0.010  0.010  0.009  0.009  0.009  0.009  0.008  0.008  0.008  0.008  0.008  0.006  0.005  0.005  0.004  0.003  0.003  0.003  0.003  0.002  0.001  0.001  0.001  0.000 |

**Notes:** The weights were calculated based on Gini index and Out-Of-Bag error.

|  | **CT** | | **MRI** | |
| --- | --- | --- | --- | --- |
| **step** | **Combination** | **AIC value** | **Combination** | **AIC value** |
| **1** | radiomics_score + sex + age + tumor_location + maximum_diameter + alcoholism&smoking | -390.7 | radiomics_score + sex + age + tumor_location + maximum_diameter + alcoholism&smoking | -356.4 |
| **2** | radiomics_score + sex + age + tumor_location + maximum_diameter | -392.1 | radiomics_score + sex + age + maximum_diameter + tumor_location | -358.2 |
| **3** | radiomics_score + sex + age + maximum_diameter | -393.3 | radiomics_score + sex + age + maximum_diameter | -359.6 |
| **4** | radiomics_score + sex + maximum_diameter | -393.3 |  |  |

**Table S3. The AIC values of different combination strategies.**

**Notes:** Radiomics_score: the outcomes given by radiomics signature; Maximum_diameter: the maximum diameter of tumor lesion; Tumor_location: the location of the tumor lesions; AIC: Akaike information criterion.

**Supplementary Formula**

**Formula S1**

CT:

P = - 3.771+ 3.618 * radiomics_score + 1.460 * sex - 0.016 * maximum_diameter

MRI:

P = - 4.013 + 3.673 * radiomics_score + 1.621 * sex - 0.019 * maximum_diameter + 0.004 * age

**Notes:** P: the possibility of being diagnosed of HEAML; Radiomics_score: the outcomes given by radiomics signature; Maximum_diameter: the maximum diameter of tumor lesion.
